# Fault Diagnosis of Wind Turbine Rotating Bearing Based on Multi-Mode Signal Enhancement and Fusion

**DOI:** 10.3390/e27090951

**Published:** 2025-09-13

**Authors:** Shaohu Ding, Guangsheng Zhou, Xinyu Wang, Weibin Li

**Affiliations:** 1 College of Electrical and Information Engineering, North Minzu University, Yinchuan 750021, China; hb249544150@gmail.com (G.Z.);; 2 College of Mechatronic Engineering, North Minzu University, Yinchuan 750021, China; 3 Ningxia Engineering Research Center for Hybrid Manufacturing Systems, Yinchuan 750021, China

**Keywords:** fault diagnosis, wind turbine, deep learning, multi-mode, feature enhancement and fusion

## Abstract

Wind turbines operate under harsh conditions, heightening the risk of rotating bearing failures. While fault diagnosis using acoustic or vibration signals is feasible, single-modal methods are highly vulnerable to environmental noise and system uncertainty, reducing diagnostic accuracy. Existing multi-modal approaches also struggle with noise interference and lack causal feature exploration, limiting fusion performance and generalization. To address these issues, this paper proposes CAVF-Net—a novel framework integrating bidirectional cross-attention (BCA) and causal inference (CI). It enhances Mel-Frequency Cepstral Coefficients (MFCCs) of acoustic and short-time Fourier transform (STFT) features of vibration via BCA and employs CI to derive adaptive fusion weights, effectively preserving causal relationships and achieving robust cross-modal integration. The fused features are classified for fault diagnosis under real-world conditions. Experiments show that CAVF-Net attains 99.2% accuracy with few iterations on clean data and maintains 95.42% accuracy in high-entropy multi-noise environments—outperforming single-model acoustic and vibration by 16.32% and 8.86%, respectively, while significantly reducing information uncertainty in downstream classification.

## 1. Introduction

According to data from the Global Wind Energy Council (GWEC), driven by the growing global demand for clean renewable energy and significant advancements in wind turbine manufacturing technology, the global installed wind power capacity has been steadily increasing [1]. Notably, while installed capacity continues to rise, the maintenance costs of wind turbines are also significantly increasing, with high failure rates being one of the primary factors [2]. The gearbox, blades, and generator are critical components of wind turbines, and rotating bearings are an indispensable part of the gearbox. As wind turbines operate over the long term in harsh outdoor environments, they are subject to abrasion from windblown sand, erosion from rain and snow, and impacts from flying debris. These external impacts or the intrusion of foreign objects may directly or indirectly affect the operation of the gearbox, thereby damaging its rotating bearings. Rotating bearing failures can lead to equipment downtime, and in severe cases, they may shorten the overall service life of the wind turbine or pose safety risks to maintenance personnel [3]. Therefore, fault diagnosis of rotating bearings under real-world operating conditions is of great significance.

Fault diagnosis of wind turbine rotating bearings is crucial for ensuring their reliable operation, a process that typically relies on the analysis of acoustic and vibration signals. Although extensive research exists, achieving high-precision and robust fault diagnosis under complex industrial noise and variable operating conditions remains a significant challenge.

Early research focused on extracting effective features from single-modality signals. For instance, Sarath et al. employed Mel-Frequency Cepstral Coefficients (MFCCs) and principal component analysis (PCA) to process acoustic signals. While this method can capture features from non-stationary signals, its diagnostic performance is highly susceptible to severe degradation under strong noise interference, and the fault information contained within a single-modality signal is inherently limited, lacking complementarity [4]. To enhance feature robustness, researchers like Tao and Xu turned to converting one-dimensional signals into two-dimensional time–frequency images for deep neural network training (e.g., CatGAN and MSCNN-BiLSTM) [5,6]. Although these methods somewhat improved feature representation, they remain fundamentally within a single-modality analysis framework, failing to overcome the bottleneck of information loss, and they possess inherent limitations in cross-modal collaboration.

To break through the ceiling of single-modality analysis, acoustic–vibration signal fusion technology emerged as a natural progression. The two-stage fusion method proposed by Shi et al. effectively compensates for the feature loss of single-modality signals in noisy environments by integrating acoustic features from multiple observation points [7]. However, such conventional fusion methods often rely on predefined or weighted fusion strategies, lacking the ability to adaptively perceive dynamic inter-modal relationships. Consequently, they struggle to consistently focus on the most critical fault information under complex and variable operating conditions.

To further optimize fusion efficacy, attention mechanisms were introduced to adaptively calibrate feature importance. The dynamic attention mechanism developed by You et al. significantly improved performance by adjusting weights based on the real-time perception of signal correlations [8]. However, its attention mechanism primarily operates within a modality or facilitates unidirectional interaction, failing to achieve deep, bidirectional cross-attention feature coordination and lacking sufficient handling of fine-grained semantic alignment. Similarly, Liu et al., in vision-language tasks, demonstrated that a bidirectional cross-attention mechanism outperforms general methods in resolving cross-modal semantic disparities [9]. This insight suggests that introducing more advanced cross-modal interaction mechanisms is key to breaking the existing performance bottleneck in acoustic–vibration fusion.

Recently, causal inference theory has provided a new perspective for fault diagnosis, offering the ability to reveal fault propagation paths and enhance model interpretability. The work by Chen and Liu et al. [10,11] demonstrates the potential of causal discovery in mining underlying mechanisms. However, existing research has not yet deeply integrated causal inference with cross-modal fusion; it fails to utilize causal relationships to guide and enhance the fusion process between acoustic and vibration signals; thus it is unable to fundamentally address the issue of unreliable fusion caused by spurious correlations.

The aforementioned methods have demonstrated the effectiveness of using acoustic signals, vibration signals, and their fused representations for fault diagnosis, as well as the capability of causal inference in discovering cross-modal causal relationships. However, most existing studies primarily rely on low-entropy clean signals collected in laboratory environments [12,13,14], which exhibit relatively low information uncertainty. Under real-world operating conditions, bearing signals are highly susceptible to interference from complex background noise, leading to increased signal entropy and heightened information uncertainty, thereby significantly challenging the robustness of existing diagnostic models. To address this practical issue from an information-theoretic perspective, this paper constructs high-entropy wind turbine rotating bearing signal datasets (Datasets A, B, C, and D) by incorporating various types of noise into public datasets [15,16], aiming to simulate realistic operational conditions and evaluate model performance in high-uncertainty environments. Furthermore, this study innovatively applies attention mechanisms (commonly used in cross-modal tasks such as text–image and acoustic–text integration) and causal inference (with its inherent advantage in causal discovery) to the field of acoustic–vibration fusion. The proposed CAVF-Net model is specifically designed to suppress interference from high-entropy noise during the feature fusion stage, and extensive fault diagnosis experiments were conducted on the constructed realistic datasets. The main contributions of this paper are as follows:

(1) Construction of wind turbine rotating bearing signal datasets that align with real-world conditions, enhancing the authenticity of the signals and highlighting the engineering application value of this study.

(2) Proposal of the CAVF-Net model, which enables feature extraction, feature enhancement, causal inference, and feature fusion of acoustic and vibration signals of wind turbine rotating bearings under real-world conditions, thereby achieving fault diagnosis.

(3) Validation, through experiments and data analysis, of the effectiveness of the proposed CAVF-Net model in feature enhancement, causal inference, feature fusion, and fault diagnosis of wind turbine rotating bearing signals.

## 2. Methods

### 2.1. Dataset Construction

This paper uses the acoustic and vibration datasets from the newly published bearing dataset by the Korea Advanced Institute of Science and Technology (KAIST) [17] as the basic research dataset (Basic Dataset). By incorporating various interfering noises collected from real-world conditions into the basic research dataset, wind turbine rotating bearing signal datasets (Datasets A, B, C, and D) that align with actual operating conditions are constructed.

#### 2.1.1. Basic Research Dataset

KAIST collected acoustic data under no-load conditions to avoid the influence of air-cooling brake noise. Acoustic and vibration data were acquired at 3010 rpm with sampling frequencies of 51.2 kHz and 25.6 kHz, respectively, under a 0 Nm load. Five types of signals were collected, including two types of inner race faults (IR) at 0.3 mm (abbreviated as FI03) and 1.0 mm (abbreviated as FI10), two types of outer race faults (OR) at 0.3 mm (abbreviated as FO03) and 1.0 mm (abbreviated as FO10), and one under a normal condition (Normal). The data sizes for the acoustic and vibration signals are 1 × 3,072,000 and 1 × 1,536,000 × 2, respectively. Details of the basic dataset are presented in Table 1 below.

#### 2.1.2. Dataset Construction

First, the KAIST dataset in the basic dataset was resampled to 25.6 kHz. Next, the acoustic and vibration signals underwent noise-mixing preprocessing. The resampled acoustic signals were mixed with white noise (WN), mechanical noise (MN), and background wind noise (BWN), while the vibration signals were mixed with white noise, vibration interference from an oil pump (VIOP), and structural vibration noise (SVN) to construct signals reflecting real-world operating conditions. The globally added white noise was generated using a normal distribution with a mean of 0 and standard deviations of 0, 0.1, 0.2, and 0.4, corresponding to low, mild, moderate, and severe interference levels, respectively. The mechanical noise, background wind noise, vibration interference from the oil pump, and structural vibration noise were all collected from real-world sources and mixed with the basic dataset in a 6:2:2 ratio. Four datasets were constructed, and they were named Datasets A, B, C, and D. Details of the basic dataset, resampled data, and the four constructed datasets are presented in Table 2.

**Figure 1:**
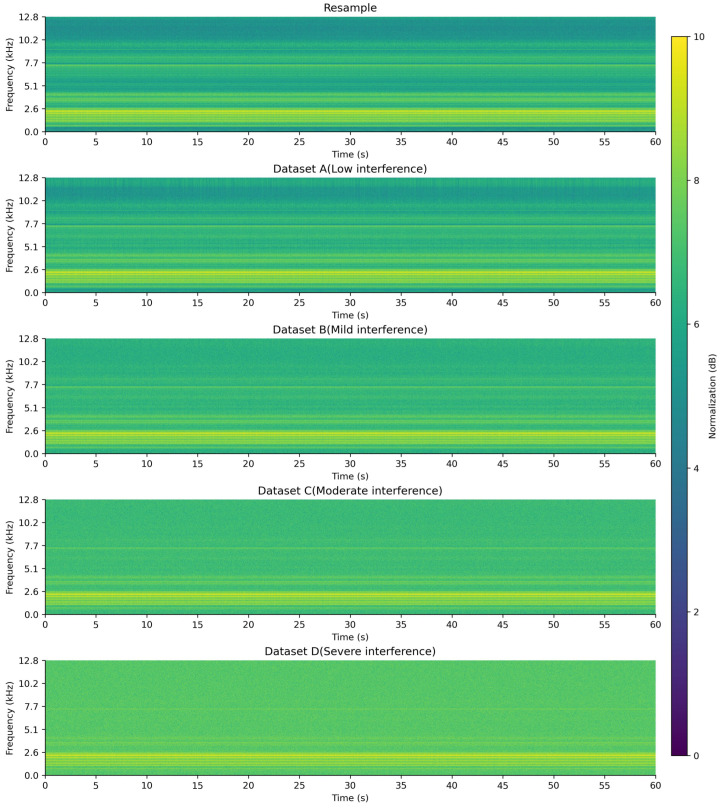
FO10 time–frequency diagram of the X-axis vibration signal under different interference conditions.

**Figure 2:**
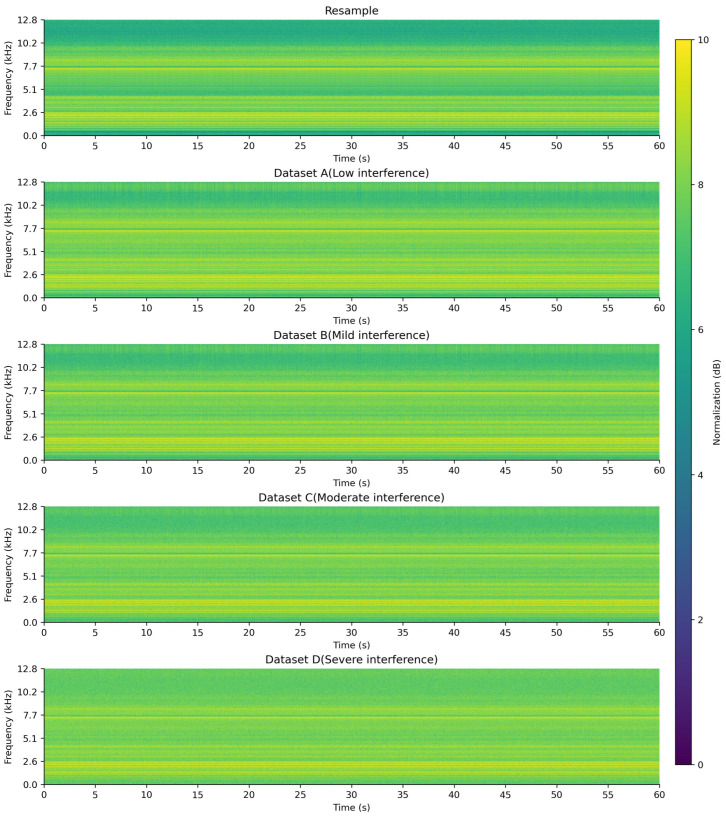
FO10 time–frequency diagram of the Y-axis vibration signal under different interference conditions.

**Figure 3:**
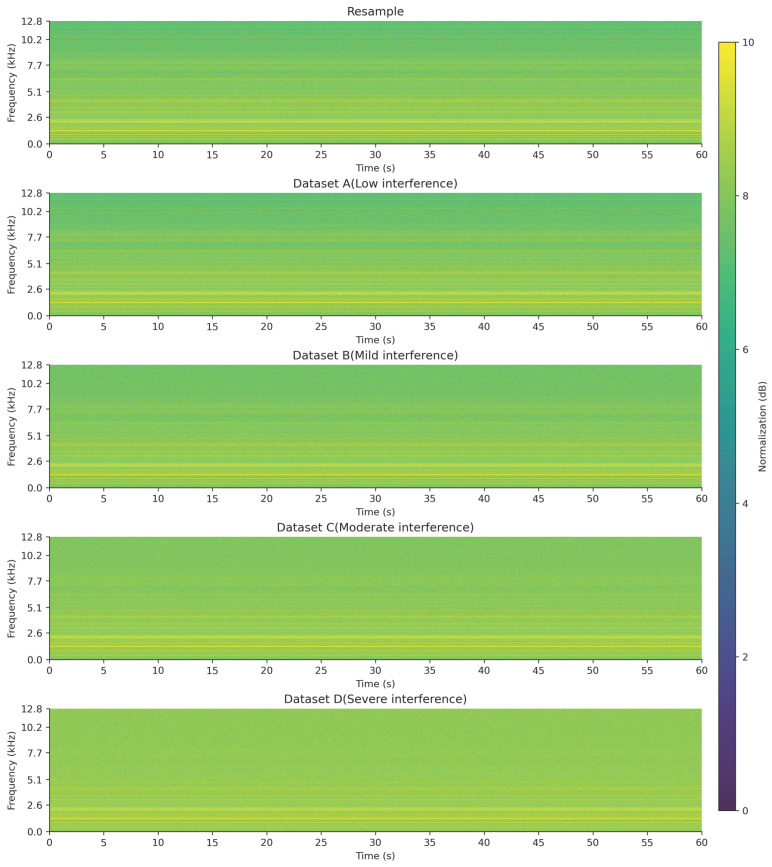
FO10 time–frequency diagram of acoustic signal under different interference conditions.

To better illustrate the results of signal construction and compare the time–frequency characteristics of signals under different interference conditions, the fault type FO10 was used as an example. The short-time Fourier transform (STFT, with a Hanning window size of 1024, a step size of 256, time as the X-axis, frequency as the Y-axis, and amplitude values converted and linearly normalized to 0–10 dB, transitioning from dark to light colors) was applied to perform time–frequency analysis on both acoustic and vibration signals, generating time–frequency spectrograms for signal visualization [18]. The time–frequency spectrograms of FO10 under different interference conditions are shown in Figure 1, Figure 2 and Figure 3.

As observed from the time–frequency spectrograms, with the increase in interference level, the amplitude values across the entire frequency band gradually converge to a uniform level, and the frequency characteristics progressively disappear, becoming unobservable.

### 2.2. CAVF-Net

CAVF-Net is a multi-modal feature fusion network designed for fault diagnosis of wind turbine rotating bearings; it is based on attention mechanisms and causal weighting. It consists of four stages: feature extraction, bidirectional cross-attention, causal inference and feature fusion, and classification.

**Figure 4:**
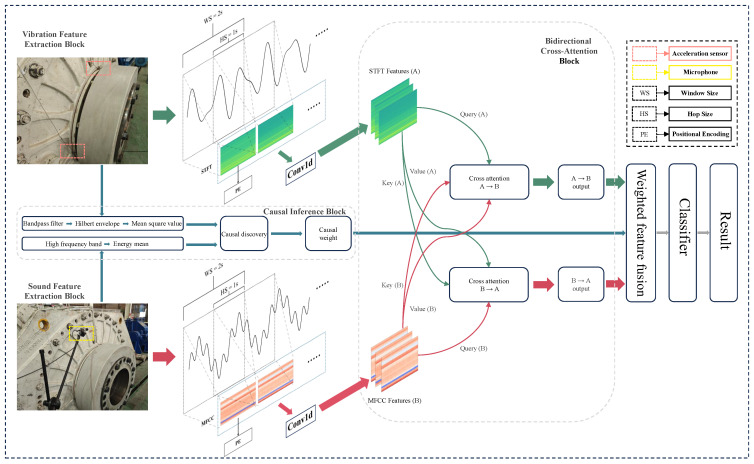
CAVF-Net overall frame structure.

First, in the feature extraction stage, the network extracts Mel-Frequency Cepstral Coefficient (MFCC) features from acoustic signals and short-time Fourier transform (STFT) features from vibration signals. These high-dimensional features are mapped to a unified dimension using one-dimensional convolution (Conv1d), and temporal information is incorporated into the sequence through sine and cosine positional encoding. Next, in the bidirectional cross-attention stage, the unified-dimension features are input into a bidirectional cross-attention module. Through a mutual query mechanism between acoustic and vibration features (i.e., bidirectional “query-key-value” computation), cross-modal information interaction and complementarity are achieved, while the attention mechanism models temporal dependencies, ultimately outputting enhanced features. Subsequently, in the causal inference and feature fusion stage, based on prior knowledge of fault types, the causal inference module calculates the contribution of acoustic and vibration signals to fault classification, determining their causal weight coefficients. These coefficients are used to perform weighted fusion of the enhanced features, generating fused features. Finally, in the classification stage, the fused features are input into a classifier module to achieve fault diagnosis. The overall framework structure of CAVF-Net is shown in Figure 4.

#### 2.2.1. Feature Extraction Stage

The feature extraction stage can be described by Equations (1)–(3), corresponding to MFCC feature extraction, STFT feature extraction, and feature normalization, respectively. The expression for MFCC feature extraction is given by Equation (1):

(1) $$F_{M}=DCT\left(\log\left(Mel\left(|DFT\left(Window\left(x_{a}\right)\right){|}^{2}\right)\right)\right),$$

where $x_{a}$ represents the acoustic signal, *Window* denotes the framing and windowing operation, *DFT* represents the discrete Fourier transform, ${\left|\right|}^{2}$ indicates the power spectrum calculation, *Mel* refers to Mel-scale filter bank filtering, *log* represents logarithmic energy compression, *DCT* denotes the discrete cosine transform, and $F_{M}$ is the MFCC feature output, with *M* indicating the MFCC.

The expression for STFT feature extraction is given by Equation (2):

(2) $$F_{s}={\left|DFT\left(Window\left(x_{v}\right)\right)\right|}^{2},$$

where $x_{v}$ is the vibration signal, $F_{S}$ is the STFT feature output, and *S* denotes STFT.

The expression for feature normalization is given by Equation (3):

(3) $$F_{x}^{\prime }=ReLU\left(Batch\left(Conv1D(F_{s},W)\right)\right),$$

where **$F_{x}$** refers to the aforementioned $F_{M}$ and $F_{S}$, *W* is the convolution kernel, *Batch* denotes the input batch size, *ReLU* is the nonlinear activation function, and $F_{x}^{\prime }$ is the normalized feature output.

#### 2.2.2. Bidirectional Cross-Attention Stage

The bidirectional cross-attention stage can be described by Equations (4)–(7), corresponding to Query, Key, and Value projections; bidirectional cross-attention computation; and feature enhancement, respectively. The expressions for the Query, Key, and Value projections are given by Equations (4) and (5):

(4) $$Q_{x},K_{x}=Linear_{x}\left(F_{x}^{\prime }\right),$$

(5) $$V_{x}=Linear_{x}^{v}\left(F_{x}^{\prime }\right),$$

where *Linear* is the linear transformation function and $Q_{x}$, $K_{x}$, and $V_{x}$ are the Query, Key, and Value projection values, respectively.

The expression for bidirectional cross-attention computation is given by Equation (6):

(6) $$\begin{array}{cc} A_{M\to S} & =\mathrm{Softmax}\left(\frac{Q_{M}K_{S}^{T}}{\sqrt{d}}\right),\\ A_{S\to M} & =\mathrm{Softmax}\left(\frac{Q_{S}K_{M}^{T}}{\sqrt{d}}\right), \end{array}$$

where *A* is the bidirectional cross-attention computation result, *Softmax* is the normalization function, and $\sqrt{d}$ is the query dimension head.

The expression for feature enhancement is given by Equation (7):

(7) $$\begin{array}{c} F_{M}^{\prime \prime }=A_{M\to S}V_{S},\\ F_{S}^{\prime \prime }=A_{S\to M}V_{M}, \end{array}$$

where $F_{x}^{\prime \prime }$ is the enhanced feature output.

#### 2.2.3. Causal Inference and Feature Fusion Stage

The causal inference and feature fusion stage can be described by Equations (8)–(13), corresponding to data preprocessing and standardization, causal discovery and causal weighting, and feature weighted fusion, respectively. The expressions for data preprocessing and standardization are given by Equations (8)–(10):

(8) $$C_{a}=T\left[HFB\left(x_{a}\right){|}^{2}\right],$$

where *HFB* is the high-frequency band channel (2000–8000 Hz), *T* is the time dimension, and $C_{a}$ is the causal acoustic signal feature.

(9) $$C_{v}=T[|Hilbert\left(BP\left(x_{v}\right)\right){|}^{2}],$$

where *BP* is the bandpass filter (100–350 Hz), *Hilbert* is the Hilbert transform for envelope extraction, and $C_{v}$ is the causal vibration signal feature.

(10) $$C_{scaled}=\frac{C_{x}-\mu }{\sigma },$$

where $C_{x}$ refers to the aforementioned $C_{a}$ and $C_{v}$, $C_{scaled}$ is the standardized feature output, μ is the mean, and σ is the standard deviation.

The expressions for causal discovery and causal weighting are given by Equations (11) and (12):

(11) G=PC(D,α=0.05),

where *D* is the feature matrix $\left(D=\left[C_{v},C_{a},Label\right]\right)$, α is the significance value, *PC* is the Peter–Clark algorithm, and *G* is the causal graph association set.

(12) $$\begin{array}{c} W_{v}^{L}=\frac{|\beta_{v}^{L}|}{|\beta_{v}^{L}\left|+\right|\beta_{a}^{L}|},\\ W_{a}^{L}=\frac{|\beta_{a}^{L}|}{|\beta_{v}^{L}\left|+\right|\beta_{a}^{L}|}, \end{array}$$

where *L* is the label (signal type), β is the regression coefficient for the fault category, *W* is the causal weight, and $W_{v}^{L}+W_{a}^{L}=1$.

The expression for feature weighted fusion is given by Equation (13):

(13) $$F^{\prime \prime \prime }=F_{M}^{\prime \prime }⊙W_{a}^{L}+F_{S}^{\prime \prime }⊙W_{v}^{L},$$

where $F^{\prime \prime \prime }$ is the weighted fused feature.

#### 2.2.4. Classifier Stage

The expression for the classifier is given by Equation (14):

(14) $$P=\mathrm{Softmax}(F^{\prime \prime \prime }),$$

where *P* is the classifier output.

## 3. Experiments and Results

### 3.1. CAVF-Net Model Training

#### 3.1.1. Feature Extraction of Acoustic and Vibration Signals

Datasets A, B, C, and D were combined to form the training dataset for the network model. This training dataset consisted of three types of signals from the four datasets, with each type corresponding to five distinct operating conditions (Normal, FI03, FI10, FO03, and FO10). To meet the requirements of the proposed CAVF-Net model for vibration and acoustic signal enhancement and fusion, the data-processing stage must ensure that the signals satisfy the following two conditions: first, providing a sufficient number of data samples to support network training, which is achieved by segmenting signals for each state using a sliding window method (total duration: 60 s, window length: 2 s, and step size: 1 s) and second, achieving temporal alignment between the acoustic and vibration signals by uniformly extracting eight time frames—40-order MFCC coefficients for the acoustic signals and 513-order STFT features for the vibration signals. The training dataset generated a total of 4 × 5 × 59 = 1180 MFCC features and 4 × 2 × 5 × 59 = 2360 STFT features. The MFCC features were temporally aligned with the two types of STFT features, resulting in 2360 corresponding acoustic–vibration feature pairs. The dataset was then split into the training and testing sets in an 8:2 ratio.

#### 3.1.2. Bidirectional Cross-Attention Model Training

**Figure 5:**
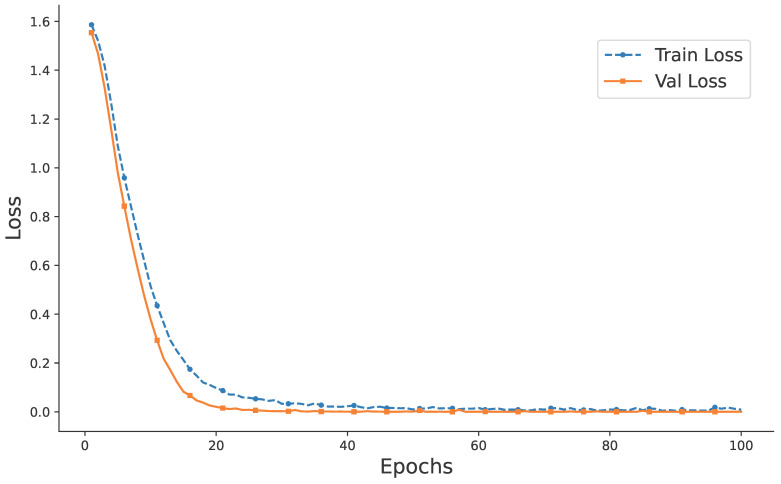
Training and validation loss rate curves.

The bidirectional cross-attention model was trained with a sampling frequency of 25.6 kHz and a batch size of 32. The cross-entropy loss function (CrossEntropyLoss) was employed, and the learning rate was set to 0.0001. The model was trained for 100 epochs. After 40 epochs, the training and validation loss curves stabilized at approximately 0.02, with minor fluctuations within a narrow range. This indicates that the network training on the input dataset was essentially complete, and the model achieved convergence. Based on general training principles, the parameters of this trained model were saved for subsequent use in noise reduction tasks for wind turbine rotating bearings. The training and validation loss curves are shown in Figure 5.

#### 3.1.3. Causal Inference

**Figure 6:**
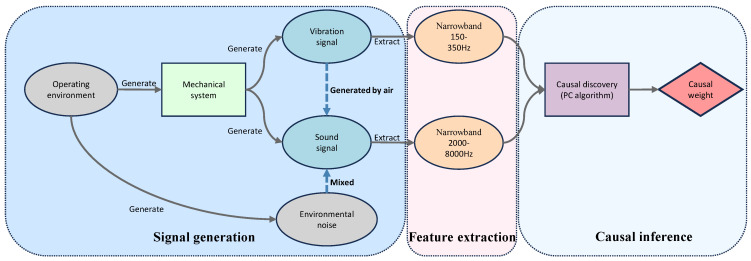
Causal inference structure.

The acoustic and vibration signals from the network model training dataset were processed using a combination of narrowband feature extraction and causal discovery to elucidate the causal relationships between these signals and signal categories, ultimately yielding causal weights. The structure of the causal inference is illustrated in Figure 6.

The acoustic signals are processed using the short-time Fourier transform to extract energy features in the 2000–8000 Hz frequency band, while the vibration signals are processed through a Butterworth bandpass filter (150–350 Hz) and the Hilbert transform to calculate the mean square value of the high-frequency energy envelope. The design logic for narrowband frequency selection is based on the publicly available parameters of the KAIST bearing dataset (bearing model: NSK 6205 DDU, rotational speed: 3010 RPM, ball diameter (d): 7.90 mm, pitch diameter (D): 38.5 mm, contact angle (θ): 0 degrees, number of balls (N): 9, shaft frequency (fs): 50.17 Hz, fundamental train frequency ($f_{FTF}$): 19.94 Hz, ball pass frequency inner race ($f_{BPFI}$): 272.07 Hz, ball pass frequency outer race ($f_{BPFO}$): 179.43 Hz, and ball spin frequency ($f_{BSF}$): 234.19 Hz) [17]. The narrowband range for vibration signals is set to 150–350 Hz to cover the fault frequencies of the inner and outer races, while a fourth-order Butterworth bandpass filter is used to eliminate low-frequency noise. For acoustic signals, based on fault diagnosis experience, impact vibrations caused by mechanical faults typically generate high-frequency acoustic signals in the 2000–8000 Hz range; thus, the narrowband range for acoustic signals is set to 2000–8000 Hz to effectively capture fault characteristics [19]. The PC algorithm, based on the Fisher Z test (significance level α = 0.05), is employed for causal discovery to construct a causal graph among variables, quantify the causal effects of features on different signal categories, and normalize these to generate weights. When the weight coefficient of a vibration or acoustic signal is higher for a specific signal type, it indicates a lower correlation of that signal with the outcome, and thus a higher weight coefficient is assigned. The causal weight coefficients are presented in Table 3.

From the data in Table 3, it can be observed that in the Normal signal type, due to the absence of specific fault impact responses, the correlation of both acoustic and vibration signals with the outcome is approximately 0.5. In the FI03 and FO10 signal types, the acoustic signals exhibit a higher correlation with the outcome compared to the vibration signals, resulting in a larger weight coefficient assigned to the vibration signals in these two signal types. Conversely, in the FI10 and FO03 signal types, the acoustic signals show a lower correlation with the outcome compared to the vibration signals, leading to a smaller weight coefficient for the vibration signals.

#### 3.1.4. Classifier Design

The fused feature has a shape of 8 × 16, which is flattened into a 128-dimensional vector. The classifier consists of a three-layer fully connected neural network with layer sizes of 128, 64, 32, and 5, respectively. The ReLU activation function is applied between layers to activate neurons, and dropout regularization is used to prevent overfitting, making the classifier suitable for multi-modal classification tasks.

### 3.2. Fault Diagnosis

#### 3.2.1. Experimental Setup

The experiments were conducted on a laptop equipped with an AMD Ryzen 7 7735H processor (3.2 GHz, 8 cores), 16 GB of DDR5 RAM (4800 MHz), and an NVIDIA RTX 4060 with 8 GB of VRAM. The operating system was Windows 11, with Python version 3.12 and CUDA version 12.8.90.

To validate the effectiveness of the proposed CAVF-Net model, nine comparative experiments were designed. The experimental schemes are detailed in Table 4.

In Table 4, Schemes 3–9 perform feature fusion after aligning the features of both modalities to a unified dimension. Schemes 7–9 additionally include a feature enhancement operation, where both features are first enhanced before being aligned to a unified dimension and then fused. Scheme 3 employs static fusion, which involves fusing features as fixed vectors without temporal information. Scheme 4 uses direct fusion, disregarding the weights between features. Scheme 5 applies a fixed weight coefficient of 0.5:0.5. Scheme 7 adopts dynamic weight coefficients, continuously adjusting the weights during the fusion process based on posterior results. Scheme 8 is an advanced version of Scheme 5 and uses the same fixed weight coefficients. Scheme 9 (CAVF-Net) is an advanced version of Scheme 6, performing feature weighting using the causal weight coefficients from Table 3 before fusion [20,21,22,23,24,25].

#### 3.2.2. Feature Analysis

To better demonstrate the extraction, enhancement, and fusion capabilities of CAVF-Net on bimodal signal features, this section visualizes the changes in the Mel-Frequency Cepstral Coefficient (MFCC) features extracted from acoustic signals and the short-time Fourier transform (STFT) features extracted from vibration signals [26]. Since this study adopts a 2 s window size for feature extraction of both signals, this section randomly selects a window from the 0–60 s range of the five signal types in Dataset A to display feature changes. To better illustrate the variations between features, the feature change graphs use a color gradient (from dark to light) to represent the energy amplitude of the features (normalized to a range of 0–10) [27,28].

**Figure 7:**
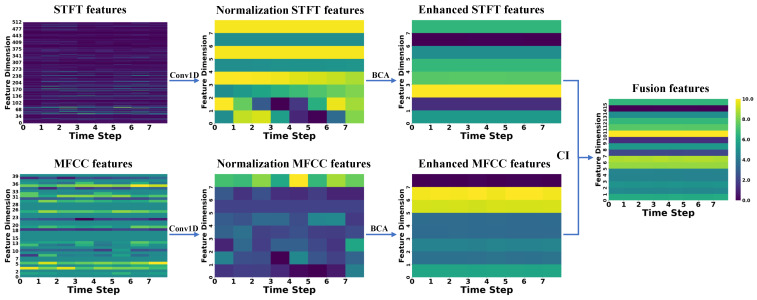
Normal-type signal.

**Figure 8:**
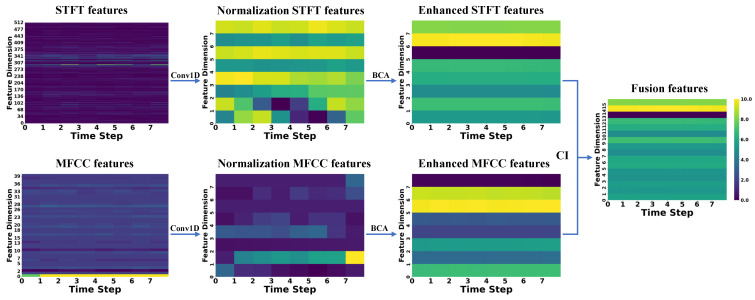
FI03-type signal.

**Figure 9:**
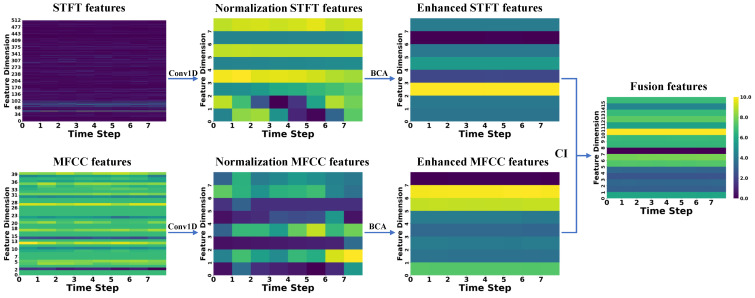
FI10-type signal.

**Figure 10:**
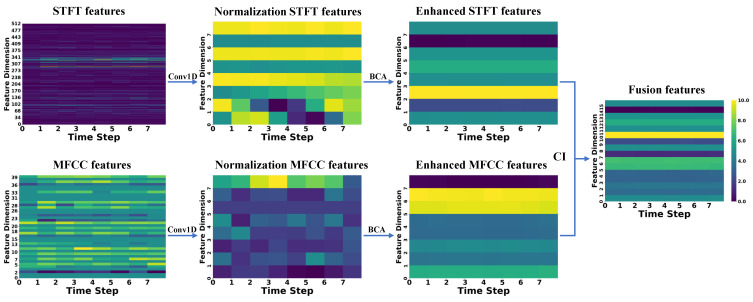
FO03-type signal.

**Figure 11:**
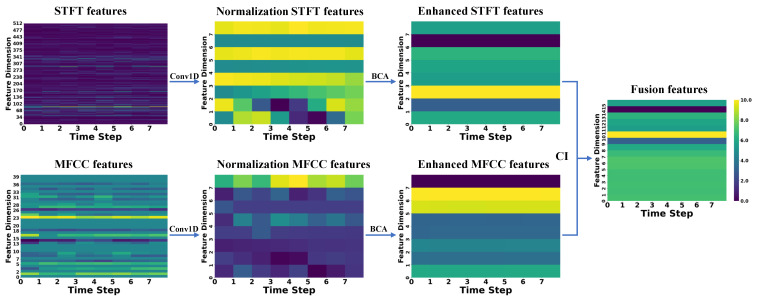
FO10-type signal.

In the figures below, the raw MFCC and STFT features directly extracted from the signals are presented first. Next, the two raw features are processed through a one-dimensional convolution (Conv1D) to align their dimensions, resulting in normalized features. Then, the normalized features are enhanced using the bidirectional cross-attention (BCA) mechanism, yielding enhanced features. Finally, the two enhanced features are weighted and fused using the causal weight coefficients derived from causal inference (CI), producing the fused features. The changes in the feature maps are shown in Figure 7, Figure 8, Figure 9, Figure 10 and Figure 11.

First, the analysis of raw features reveals that a larger number of feature coefficients were designed during feature extraction to extract more feature information, leading to weak significance in certain feature dimensions. These weakly significant features severely impact the accuracy of fault diagnosis. Second, the analysis of normalized features shows that after normalization, the feature dimensions are standardized to eight, effectively controlling the proportion of weakly significant dimensions in the feature map. However, the differences between STFT and MFCC features remain substantial. Next, the analysis of enhanced features indicates that after enhancement through the bidirectional cross-attention mechanism, the energy amplitude of the features undergoes significant changes. The bidirectional cross-attention mechanism facilitates information interaction between features, resulting in MFCC features being influenced by STFT features and achieving overall enhancement, while STFT features experience an overall reduction. Finally, by incorporating causal weight coefficients, effective fusion of the two modal features is achieved. This ensures that the classification results are not dominated by a single modality when it is more prominent in certain feature dimensions and that fault-related features are overlooked when certain dimensions exhibit weaker significance. After multi-modal feature fusion, the two modal features complement each other, making the signal features more distinct and enabling the classifier to perform fault classification more easily.

#### 3.2.3. Accuracy Analysis

##### Comparative Experiments for Schemes 1–6

**Figure 12:**
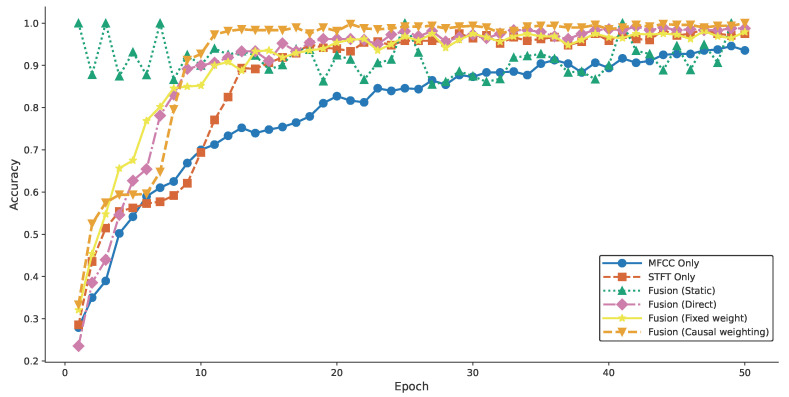
Classification accuracy experiment results for Schemes 1–6.

Comparative experiments for Schemes 1–6 were conducted to evaluate the differences in fault diagnosis accuracy between single-modality and multi-modal feature approaches, as well as to validate the impact of causal weights on feature fusion [29]. The classification accuracy for Schemes 1–6 is shown in Figure 12.

Figure 12 shows that fault diagnosis accuracy using vibration signal features was higher than that using acoustic signal features, indicating that low-frequency features are less affected by interference in harsh environments. However, the accuracy of both single-modality approaches was significantly lower than that of the fused feature schemes, demonstrating the practical engineering value of cross-modal feature fusion for fault diagnosis. Different fusion schemes also resulted in varying accuracies. The static fusion scheme with SVM achieved high initial accuracy but failed to converge through iterations, showing clear limitations compared to other schemes. The direct fusion scheme had the lowest initial accuracy but exhibited a faster convergence rate than other schemes, except for the causal weight scheme. This suggests that high-energy acoustic signal features initially interfered with low-energy vibration signal features, negatively impacting fault diagnosis. The fixed-weight fusion scheme mitigated the issues of direct fusion, achieving the second-highest initial accuracy among Schemes 1–6. However, due to the limitations of fixed weights, it exhibited slower convergence and lower accuracy in later iterations, indicating that fixed weight coefficients constrained the influence of both features on classification results, leading to mutual restriction and preventing high accuracy. The causal weight fusion scheme achieved the best relative performance, with the highest initial accuracy, the earliest attainment of high accuracy, and stable performance in subsequent iterations, confirming the effectiveness of causal weight coefficients in acoustic–vibration fusion.

##### Comparative Experiments for Schemes 6–9

**Figure 13:**
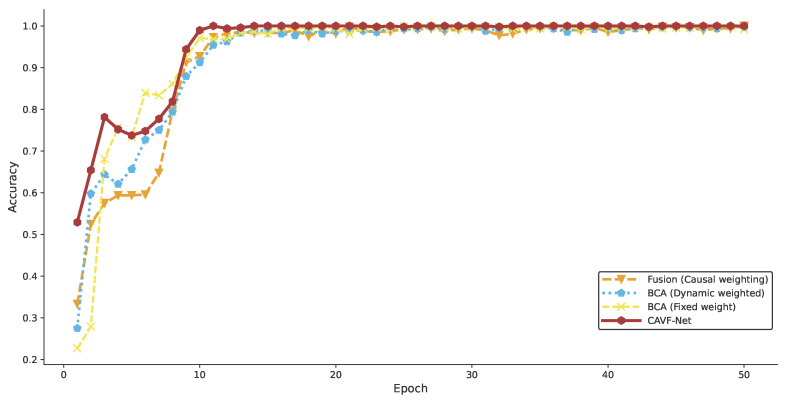
Classification accuracy experiment results for Schemes 6–9.

Comparative experiments for Schemes 6–9 were conducted to validate the effectiveness of feature enhancement using the bidirectional cross-attention mechanism for fault diagnosis and to compare the fault diagnosis accuracy of schemes with feature enhancement but different fusion strategies. The classification accuracy for Schemes 6–9 is shown in Figure 13.

Figure 13 shows that the proposed CAVF-Net scheme achieved the best performance, consistent with Scheme 6, which used causal weight coefficients for fusion without feature enhancement. Both schemes achieved relatively high initial accuracy, but the CAVF-Net scheme, with feature enhancement, showed significant improvement and was the first to reach high accuracy in subsequent iterations. Comparing Schemes 6 and 9 confirms the effectiveness of feature enhancement via the bidirectional cross-attention mechanism in acoustic–vibration fusion. Although Schemes 7 and 8 also employed feature enhancement, their use of alternative fusion strategies resulted in high accuracy in later iterations, but the CAVF-Net scheme outperformed both, achieving higher initial accuracy in early fault diagnosis stages. This performance surpassed schemes with fixed or adaptive weights, further validating the effectiveness of causal weight coefficients in acoustic–vibration fusion.

##### Mean Accuracy Comparison

Finally, a mean accuracy table was compiled to intuitively compare the fault diagnosis performance of different schemes [30]. The mean accuracy is presented in Table 5.

As shown in Table 5, the proposed CAVF-Net scheme achieves the highest average accuracy of 95.42%. The average accuracy using only acoustic signals or vibration signals is relatively low, at 79.10% and 86.56%, respectively. The average accuracies of Schemes 3–6, which use fused features, are 91.48%, 89.89%, 89.94%, and 92.12%, respectively. Among these, the scheme employing causal weights outperforms the direct fusion and fixed-weight fusion schemes by approximately 2.22%. In Schemes 7–9, which further incorporate feature enhancement, Scheme 8 surpasses its baseline Scheme 5 with a 3.72% improvement in accuracy; the CAVF-Net scheme outperforms its baseline Scheme 6 with a 3.3% improvement; Scheme 7 achieves a relatively lower accuracy of 92.94% but still outperforms other schemes without feature enhancement. The CAVF-Net scheme’s average accuracy is 16.32% and 8.86% higher than the single-modal Schemes 1 and 2, respectively.

#### 3.2.4. Comparative Analysis of Existing Methods

**Figure 14:**
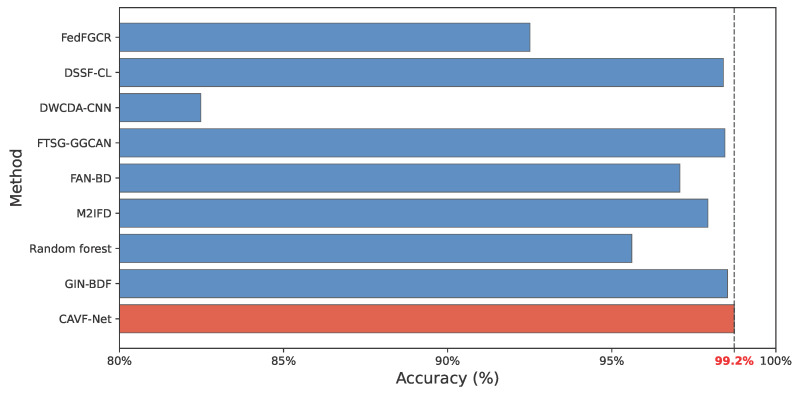
Comparison diagram of accuracy of existing methods.

To validate the superiority of the proposed CAVF-Net, this study selects fault diagnosis methods based on the KAIST dataset as benchmarks for comparison. Literature analysis reveals that existing methods typically use raw signals for diagnosis, with significant variations in input modalities and network architectures. To ensure rigorous comparisons, this section employs raw signals as model inputs. The comparative experiments aim to verify the performance advantages of CAVF-Net from two dimensions: diagnostic accuracy and training efficiency. In terms of accuracy, the highest accuracy of different methods on the same test set is compared; in terms of efficiency, the relative number of iterations required to achieve comparable performance is compared. Additionally, this section takes the results of CAVF-Net as the baseline and mainly analyzes the relationship between the number of iterations and the results. Efficiency is calculated relative to the iteration rounds of CAVF-Net (benchmark = 1.0×). A higher value indicates that a method requires more iterations and thus has lower efficiency. For methods where iteration counts are unavailable or unnecessary (e.g., non-iterative models), the table denotes these as N/A and reports only their highest accuracy results. The comparison of methods is presented in Table 6 and Figure 14.

The results in Table 6 and Figure 14 demonstrate that the proposed CAVF-Net model significantly outperforms existing methods of the same type in both accuracy and efficiency, achieving an optimal accuracy of 99.20% with only 50 iterations using raw signals. In contrast, while GIN-BDF achieves an accuracy of 98.99%, it requires four input modalities, increasing computational costs. FTSG-GGCAN and DSSF-CNN both attain accuracies above 98%, but their iteration efficiencies are lower than that of the proposed method. DWCDA-CNN achieves only 82.54% accuracy, making it unsuitable for high-precision scenarios. FedFGCR requires 300 iterations to reach 92.82% accuracy, indicating lower efficiency. Finally, the three methods without iteration data or requiring no iterations all exhibit maximum fault diagnosis accuracies lower than that of the proposed method.

The results demonstrate that the proposed CAVF-Net scheme achieves the greatest improvement in accuracy for fault diagnosis on public datasets, validating its ability to preserve cross-modal signal features. This highlights the network’s high engineering application value and its suitability for fault diagnosis.

## 4. Conclusions

First, this study collected and simulated environmental noise under real-world operating conditions of wind turbine rotating bearings and integrated it with public rotating bearing datasets to construct acoustic and vibration signal datasets that align with actual operating conditions. Additionally, acoustic and vibration signals from actual wind turbine gearbox bearings were collected to build a validation dataset. Second, CAVF-Net was designed to extract Mel-Frequency Cepstral Coefficient (MFCC) features from acoustic signals and short-time Fourier transform (STFT) features from vibration signals, followed by feature enhancement and fusion. Then, through experiments and data analysis, the effectiveness of CAVF-Net in fault diagnosis was verified. The network effectively preserves fault features in acoustic and vibration signals, achieving an accuracy of 99.2% on a noiseless public dataset; specifically, in high-entropy environments composed of multiple noise sources, it maintains an average fault diagnosis accuracy of 95.42%, significantly reducing the information uncertainty faced by downstream classification tasks. This enables the acoustic–vibration fusion fault diagnosis of rotating bearings under actual working conditions. In conclusion, the proposed CAVF-Net model demonstrates excellent anti-interference and feature preservation capabilities in high-uncertainty environments, exhibiting high engineering application value and theoretical significance and holding substantial positive implications for advancing fault diagnosis of wind turbine rotating bearings.

## Figures and Tables

**Table 1:** Basic research dataset.

Dataset	Data Type	Frequency (kHz)	Load	Location	Numbers of Data Files (.mat)
					Normal, FI03, FI10, FO03, and FO10
Basic	Acoustic	51.2	0	A	3,072,000 × 5
	Vibration	25.6	0	A (X axis)	1,536,000 × 5
				A (Y axis)	1,536,000 × 5

**Table 2:** Dataset Content.

Dataset	Frequency (kHz)	Data Type (Acoustic and Vibration)	Contain Types (FI03, FI10, FO03, FO10, and Normal)	Duration (s)	Interference
Basic	51.2 and 25.6	All	All	5 × 60 × 3	None
Resample	25.6	All	All	5 × 60 × 3	None
Dataset A	25.6	All	All	5 × 60 × 3	Acoustic (MN and BWN),
					Vibration (VIOP and SVN)
Dataset B	25.6	All	All	5 × 60 × 3	Acoustic (0.1 WN, MN, and BWN),
					Vibration (0.1 WN, VIOP, and SVN)
Dataset C	25.6	All	All	5 × 60 × 3	Acoustic (0.2 WN, MN, and BWN),
					Vibration (0.2 WN, VIOP, and SVN)
Dataset D	25.6	All	All	5 × 60 × 3	Acoustic (0.4 WN, MN, and BWN),
					Vibration (0.4 WN, VIOP, and SVN)

**Table 3:** Causal weight coefficient.

Signal Type	Vibration Signal	Acoustic Signal
Normal	0.4559	0.5441
FI03	0.8122	0.1878
FI10	0.1547	0.8453
FO03	0.3785	0.6215
FO10	0.8924	0.1076

**Table 4:** Control experiment scheme.

Experimental Scheme	Data Type (Acoustic, Vibration)	Feature Enhancement	Feature Fusion Mode
Acoustic signal only (1)	Acoustic	None	None
Vibration signal only (2)	Vibration	None	None
Multi-modal + SVM (3)	**All**	None	Static feature fusion
Multi-modal + early fusion (4)	**All**	None	Direct feature fusion
Multi-modal + late fusion (5)	**All**	None	Fixed weight fusion
Multi-modal + causal inference (6)	**All**	None	**Causal weighting fusion**
Multi-modal + BCA + gated fusion (7)	**All**	**Bidirectional Cross-Attention**	Dynamic weighted feature fusion
Multi-modal + BCA + late fusion (8)	**All**	**Bidirectional Cross-Attention**	Fixed weight fusion
**CAVF-Net (9)**	**All**	**Bidirectional Cross-Attention**	**Causal weighting fusion**

**Table 5:** Average accuracy.

Experimental Scheme	Average Accuracy (%)
Acoustic signal only (1)	79.10
Vibration signal only (2)	86.56
Multi-modal + SVM (3)	91.48
Multi-modal + Early Fusion (4)	89.89
Multi-modal + Late Fusion (5)	89.34
Multi-modal + Causal Inference (6)	92.12
Multi-modal + BCA + Gated Fusion (7)	92.94
Multi-modal + BCA + Late Fusion (8)	93.66
**CAVF-Net (9)**	**95.42**

**Table 6:** Comparison of existing methods.

Method	Input Modes	Epochs	Efficiency	Accuracy (%)	Accuracy Rank
**CAVF-Net (Baseline)**	**Vibration and Acoustic**	**50**	**1.0×**	**99.20**	**Superior**
GIN-BDF [31]	Vibration, Temperature, Acoustic, and Current	100	2.0×	98.99	High
Random forest [32]	Vibration, Temperature, and Current	N/A	N/A	96.00	Moderate
M2IFD [33]	Vibration, Current	N/A	N/A	98.38	High
FAN-BD [34]	Vibration, Current	N/A	N/A	97.50	High
FTSG-GCCAN [35]	Vibration	100	2.0×	98.91	High
DWCDA-CNN [36]	Current	200	4.0×	82.54	Low
DSSF-CL [37]	Vibration	100	4.0×	98.66	High
FedFGCR [38]	Vibration	100	6.0×	92.82	Moderate

## Data Availability

The data presented in this study are available upon request from the corresponding author.

## Abbreviations

The following abbreviations are used in this manuscript:

CAVF-Net	Causal Acoustic–Vibration Fusion Network
BCA	Bidirectional Cross-Attention
MFCC	Mel-Frequency Cepstral Coefficient
STFT	Linear Short-Time Fourier Transform
